# Effects of Immediate Dentin Sealing and Quercetin on the Fracture Strength of Ceramic Inlays in Premolars of Daibetics

**DOI:** 10.1016/j.identj.2026.111089

**Published:** 2026-09-04

**Authors:** Fereshteh Shafiei, Zahra Jowkar, Samaneh Sarrafi, Mahshid Mohammadi-Bassir

**Affiliations:** a Oral and Dental Disease Research Center, Department of Operative Dentistry, School of Dentistry, Shiraz University of Medical Sciences, Shiraz, Iran; b Department of Operative Dentistry, School of Dentistry, Shiraz University of Medical Sciences, Shiraz, Iran; c Department of Operative Dentistry, School of Dentistry, Shahed University, Tehran, Iran

**Keywords:** Diabetes mellitus, Dentin bonding, Fracture resistance, Immediate dentin sealing, Quercetin

## Abstract

**Aim:**

This in vitro study evaluated the effects of immediate dentin sealing (IDS) versus delayed dentin sealing (DDS), dentin pretreatment with quercetin (Qu), and their combination on the fracture strength (FS) of diabetic and non-diabetic premolars restored with ceramic inlays after thermomechanical aging.

**Methods:**

Forty-eight extracted premolars from diabetic individuals and forty-eight from non-diabetic individuals were collected and prepared for standardized MOD lithium disilicate ceramic inlays. Each category of teeth was divided into four groups according to the cementation technique (IDS or DDS) and the presence or absence of Qu pretreatment during inlay cementation. For IDS, the same adhesive was applied in combination with a flowable composite. All restored premolars underwent thermomechanical aging followed by FS testing under axial compression. Data were analyzed using three-way ANOVA and t-tests (*P* = .05).

**Results:**

Technique, Qu treatment, diabetes mellitus (DM), and the interactions between DM and Qu (*P* = .004) and between DM and technique (*P* = .021) were significant. The interaction among all three factors and the interaction between technique and Qu were not significant. In all groups except DDS/Qu, diabetic premolars exhibited lower FS than non-diabetic premolars (*P* < .001). IDS resulted in significantly higher FS in both tooth types (*P* < .001). The positive effect of Qu pretreatment was observed only in diabetic premolars (*P* < .001).

**Conclusion:**

In diabetic premolars, IDS and Qu pretreatment substantially enhanced the FS of teeth restored with ceramic inlays, with the greatest improvement observed when both were combined. In non-diabetic premolars, a significant improvement was observed only with IDS.

**Clinical relevance:**

Diabetes-related changes in dentin may compromise the performance of adhesive restorations. Understanding the effects of dentin sealing techniques and the potential benefits of Qu may help improve the durability of indirect restorations in patients with diabetes.

## Introduction

Following extensive removal of deep caries in posterior teeth, indirect adhesive restorations have become the preferred option, as they facilitate the creation of proper proximal contours, accurate occlusal morphology, and offer favorable mechanical properties and marginal adaptation.^1^^,^^2^ However, in large cavities with substantial dentin exposure, achieving a reliable dentin bond remains a significant challenge when using the indirect approach.^3^ Adhesive cementation in indirect restorations is influenced by both the resin cement system and the properties of the underlying dental substrate, which together affect the integrity and durability of the adhesive interface.^4^ Since durable bonding is essential for the long-term success of these restorations, optimizing this step is critical.^5^

As a common clinical approach, delayed dentin sealing (DDS) during final cementation, following a temporization period with possible different contaminants is conducted.^6^^,^^7^ To overcome this issue, immediate dentin sealing (IDS) has been proposed as a strategy to bond to freshly cut dentin.^8^ Beyond reducing postoperative sensitivity and marginal gap formation,^8^^,^^9^ IDS has been shown to enhance bond strength, improve the mechanical performance of indirect restorations, and reinforce the prepared tooth structure.^3^^,^^10^, ^11^, ^12^ For these benefits to be realized, a strong and durable bond between the dentin, adhesive, and resin cement is essential.^3^

In diabetic patients, alterations in the physicochemical properties and structural integrity of dentin have been documented.^3^ Hyperglycemia accelerates protein glycation, which disrupts normal mineralized matrix formation.^13^^,^^14^ As a result, reduced peritubular dentin calcification and increased tubular density and diameter may lower dentin microhardness and negatively affect the bonding performance of adhesive systems.^13^, ^14^, ^15^, ^16^ Additionally, hyperglycemia and oxidative stress enhance the activity of matrix metalloproteinases (MMPs), particularly MMP-2 and MMP-9.^17^ These enzymes degrade exposed collagen fibrils within the hybrid layer, contributing to long-term deterioration of the resin–dentin bond.^18^

Quercetin (Qu) is a naturally occurring flavonoid present in various fruits and vegetables, including apples, cranberries, onions, and peppers. It exhibits a wide range of therapeutic and health-promoting effects, such as antioxidant, antimicrobial, anti-inflammatory, anticancer, and antidiabetic activities.^19^ In dentistry, Qu functions as an MMP inhibitor and a collagen cross-linking agent due to its high polyphenol content.^19^, ^20^, ^21^ These properties may help enhance dentin bond strength and improve bond durability.^19^^,^^22^ However, its potential benefits specifically for diabetic dentin have not yet been elucidated.

Some studies have shown that applying IDS can increase the fracture strength (FS) of teeth restored with indirect restorations.^10^^,^^11^ In addition, incorporating a polyphenolic compound such as proanthocyanidin into the adhesive system used for IDS has been reported to significantly enhance the fracture resistance of premolars restored with indirect restorations.^3^ However, the influence of Qu treatment during IDS on the strength of restored diabetic teeth has not yet been investigated. It was postulated that combining Qu with IDS in ceramic inlay restorations might reinforce the fracture strength of restored diabetic teeth. Since this topic remains unexplored in the existing literature, the present study aimed to examine this question. The null hypothesis was that IDS and Qu, applied individually or in combination, would have no effect on the FS of ceramic-inlay-restored diabetic premolars compared with non-diabetic teeth following thermomechanical aging.

## Materials and method

This experimental study used 48 intact maxillary premolars extracted from diabetic patients and another 48 sound maxillary premolars collected from non-diabetic individuals, all free of caries, fractures, and enamel defects. The study protocol was approved by the Research and Ethics Committee of Shiraz University of Medical Sciences (IR.SUMS.DENTAL.REC.1405.005), and all procedures followed the ethical principles outlined in the Declaration of Helsinki. Teeth were extracted for orthodontic purposes, and written informed consent was obtained after participants were fully informed about the aims of the study and the intended use of their extracted teeth. All experimental procedures were carried out by a single trained operator. To minimize bias, the specimens were coded, and the FS testing was performed by an investigator blinded to these codes. After extraction, the teeth were debrided using a periodontal curette and stored in 0.5% chloramine-T at 4 °C for a maximum of one month prior to use.

### Sample size calculation

**Fig. 1:**
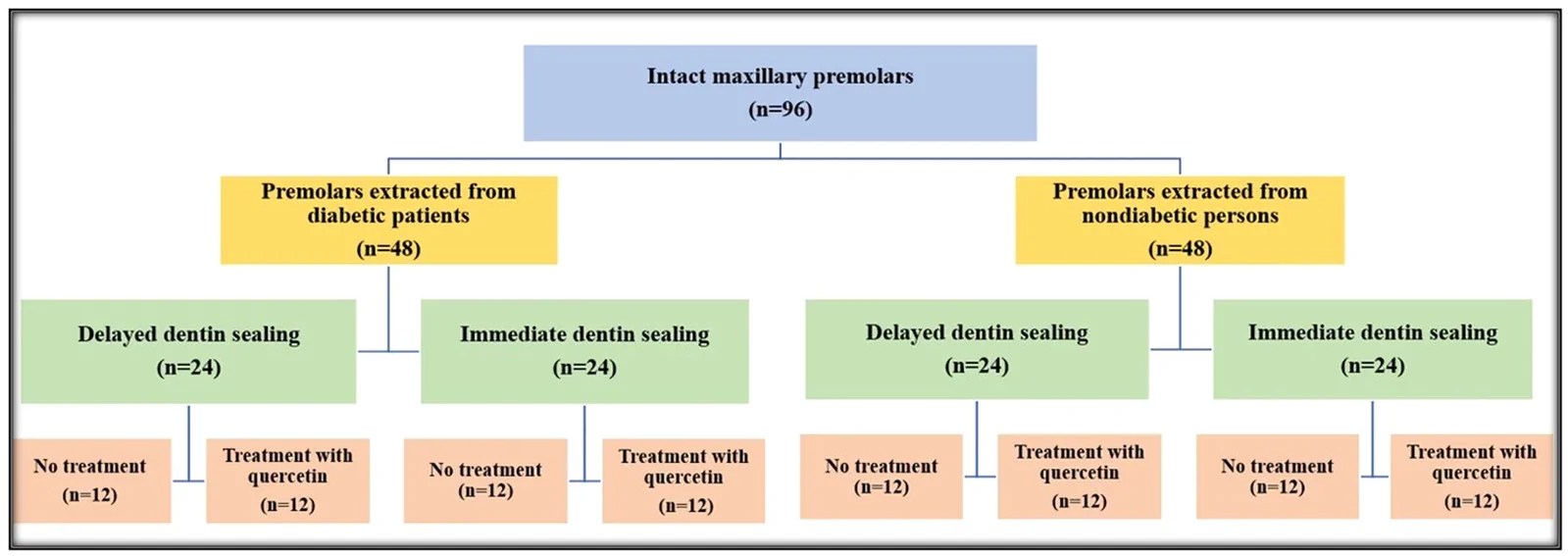
Study design flowchart. Ninety-six intact maxillary premolars were allocated into two primary groups based on donor status (diabetic, *n* = 48; non-diabetic, *n* = 48). Each group was subdivided by sealing technique into Delayed Dentin Sealing (DDS) and Immediate Dentin Sealing (IDS) (*n* = 24 each). Final subgroups (*n* = 12) were defined by the presence or absence of 1% quercetin treatment.

For this investigation, eight experimental groups were established based on tooth condition (diabetic or non-diabetic), dentin sealing approach (IDS or DDS), and the application of Qu (with or without Qu). Before conducting the experiment, the sample size was determined using data from a previous study.^3^ In that study, the comparison between the IDS/PA and DDS/PA groups yielded a pooled standard deviation of 124 N and an effect size (Cohen’s d) of 1.93. Using G*Power software (G*Power 3.1; Heinrich Heine University, Düsseldorf, Germany) with an alpha of 0.05 and a power of 80%, the minimum number of specimens needed was calculated to be five per group. To accommodate the three-factor experimental design and account for any potential specimen loss during thermomechanical aging, each group ultimately included 12 teeth. Figure 1 presents a flowchart summarizing the study design, detailing the allocation of extracted teeth according to donor diabetic status, dentin sealing technique, and Qu treatment.

A total of 48 intact maxillary premolars extracted from diabetic patients with no additional systemic conditions were collected. The donors were 35 to 55 years old and had been diagnosed with type 2 diabetes mellitus (DM) for 5 to 15 years, as verbally confirmed by each patient. The diabetic status of the patients was determined based on medical history and verbal confirmation at the time of extraction; however, laboratory confirmation such as HbA1c or fasting blood glucose values was not available. An equivalent number of sound premolars (n = 48) were obtained from non-diabetic individuals aged 35 to 55 years to achieve age comparability with the diabetic group. All selected teeth were comparable in size and free of cracks or structural defects. After cleaning, they were disinfected in 0.5% chloramine solution and stored in distilled water at 4 °C.

Before embedding the teeth in self-curing acrylic resin up to 1 mm below the cementoenamel junction (CEJ), the roots were coated with a 0.2 to 0.3 mm layer of melted wax. This wax layer was then replaced with elastomeric impression material to simulate the periodontal ligament.^23^ Each tooth was positioned with its long axis perpendicular to the base of the acrylic cylinder.

### Inlay preparations

A putty impression was taken of each premolar before cavity preparation. Standard MOD inlay cavities were prepared using a round-ended, 6° tapered fissure diamond bur, which was replaced after every four preparations. The cavity design followed these dimensions: occlusal depth of 2.5 mm, occlusal isthmus width equal to one-third of the intercuspal distance, proximal box width equal to one-third of the buccolingual dimension, gingival floor depth of 1.5 mm, and an axial wall height of 2 mm. All internal line angles were rounded.^23^ The cervical margin was positioned 1 mm coronal to the CEJ in enamel. The axial walls were prepared with slight divergence to allow proper insertion and removal of the inlays. All cavity dimensions were verified using a digital caliper and a periodontal probe.

### Impression and inlay fabrication

**Fig. 2:**
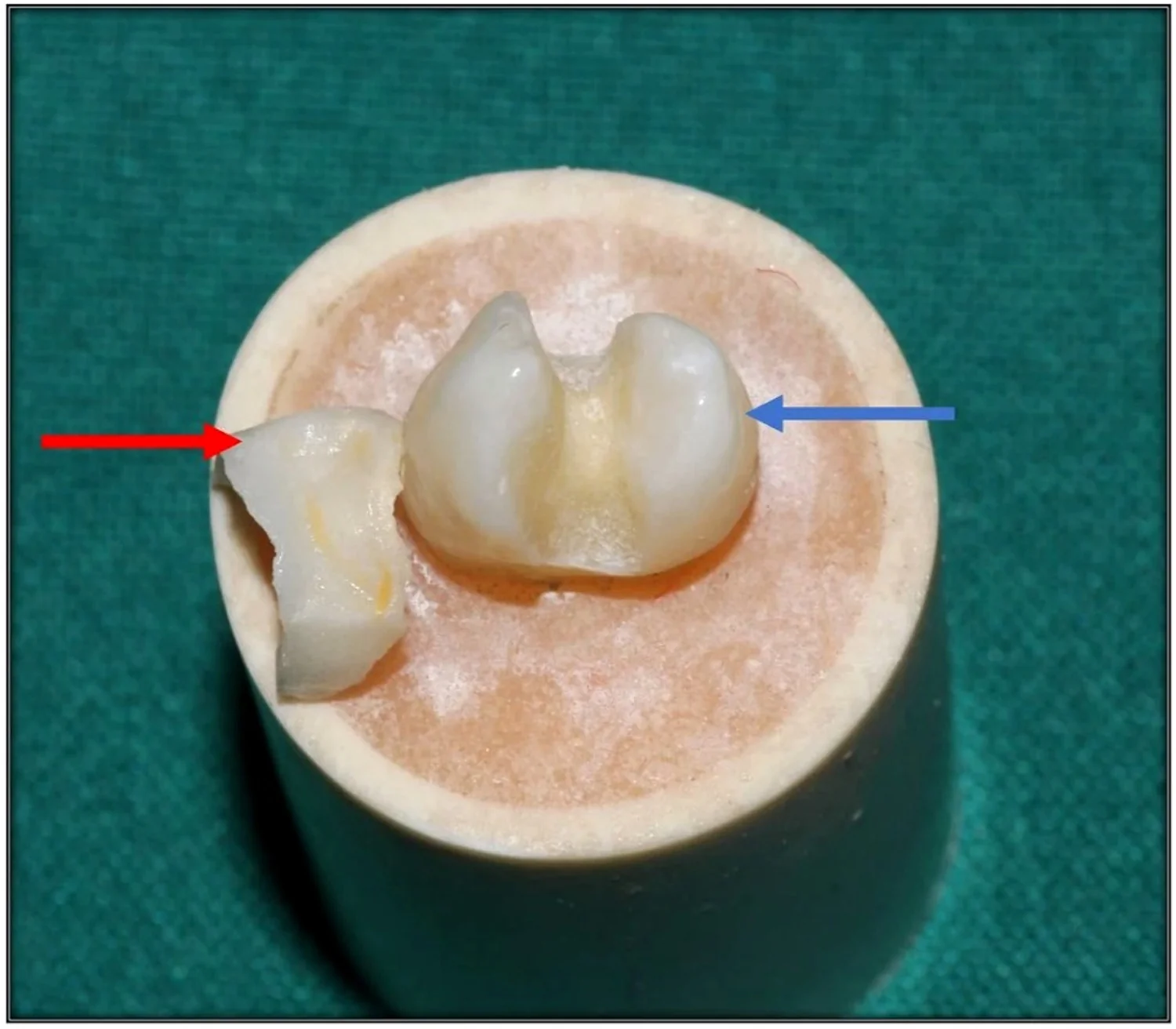
Specimen preparation and restoration components. A representative image illustrating a standardized cavity-prepared maxillary premolar (blue arrow) and the corresponding fabricated ceramic inlay restoration (red arrow) prior to the cementation procedure. The image demonstrates the internal adaptation and the conservative preparation design used for all experimental groups.

A final impression was taken using Zhermack Hydrorise Putty and Hydrorise Light Body (Zhermack S.p.A., Badia Polesine, Italy) and sent to the dental laboratory for processing. Lithium disilicate inlays (IPS e.max Press, Ivoclar Vivadent, Schaan, Liechtenstein) were fabricated by an experienced dental technician following the manufacturer’s guidelines. Figure 2 shows a representative maxillary premolar after standardized inlay cavity preparation together with the fabricated indirect inlay restoration prior to cementation.

### Groups descriptions

The teeth were first classified according to donor diabetic status (diabetic or non-diabetic). After specimen preparation, a computer-generated randomization sequence was used to allocate the specimens equally among the four experimental groups within each category (n = 12 per group), thereby ensuring balanced group sizes and minimizing allocation bias. The operator performing the FS testing was blinded to the group allocation to minimize measurement bias. The diabetic and non-diabetic specimens were then assigned to the following four experimental groups:

#### Group 1 (DDS, delayed dentin sealing)

Delayed dentin sealing was performed. After taking the final impression, provisional restorations were made from bis-acryl resin (Protemp 4, 3M ESPE, USA) and cemented with a temporary luting material (Temp Bond Clear, Kerr, USA).

#### Group 2 (DDS/Qu, delayed dentin sealing/Qu treatment)

Delayed dentin sealing with additional Qu treatment. The procedure followed the same steps as Group 1, except that during final cementation, the acid-etched dentin was treated with Qu solution for 60 seconds. Details of the Qu application are described in the final cementation protocol.

#### Group 3 (IDS, immediate dentin sealing after inlay preparation)

Immediate dentin sealing was carried out after cavity preparation. Dentin surfaces were etched with 37% phosphoric acid for 10 seconds, rinsed for 20 seconds, and gently air-dried. All-Bond Universal (ABU; Bisco Inc., Schaumburg, IL, USA) was applied in two coats, each scrubbed for 15 seconds, followed by gentle air drying for 10 to 15 seconds and light curing for 10 seconds using a LED unit (Litex™, Dentamerica, CA, USA) with an output of 1200 mW/cm².

A thin layer of flowable resin composite (≈1 mm; Aelite Flowable Composite, Bisco, Schaumburg, IL, USA) was placed over the sealed dentin and light-cured for 40 seconds.^24^ Glycerin gel was then applied to prevent oxygen inhibition, and an additional 10-second light cure was performed. This layer prevented bonding to the provisional material. Provisional restorations were fabricated and temporarily cemented as described for the DDS groups.

#### Group 4 (IDS/Qu, immediate dentin sealing/ Qu treatment)

Immediate dentin sealing was performed with Qu treatment in Group 4. The procedure followed the same steps as Group 3, except that the acid-etched dentin was treated with a 1% Qu solution for 60 seconds. A 1% Qu solution was selected based on previous reports demonstrating that this concentration provides effective inhibition of dentin MMPs and promotes collagen cross-linking without adversely affecting the adhesive interface or resin polymerization.^21^ The solution was prepared by dissolving 1 g of Qu powder (Sigma-Aldrich, St. Louis, MO, USA) in pure ethanol and heating in a water bath at 37 °C for 15 minutes.^21^ The solution was actively applied to the dentin with a microbrush and gently air-dried.^21^

### Adhesive final cementation

After a two-week storage period, the provisional restorations were removed, and the tooth surfaces were cleaned using 50 µm aluminum oxide airborne-particle abrasion (5 seconds at 1.5 cm distance and 2 bar pressure), followed by 15 seconds of phosphoric acid etching, rinsing, and drying. A thin layer of adhesive resin was then applied without light curing to allow complete seating of the final restoration.^24^ The inlay was tried in and its fit verified.

The internal surface of each inlay was etched with 5% hydrofluoric acid for 20 seconds, rinsed with water, and ultrasonically cleaned in distilled water for 5 minutes. After drying, a silane coupling agent was applied and allowed to react for 60 seconds, followed by a layer of HEMA-free porcelain bonding resin (Porcelain Bonding Resin, Bisco, Schaumburg, IL, USA) without polymerization.

In the DDS groups, enamel and dentin were etched with 37% phosphoric acid for 15 seconds, rinsed, dried, and covered with two coats of All-Bond Universal (ABU). In the IDS groups, the sealed dentin surface was rewetted using porcelain bonding resin.^24^ In both approaches, the adhesive layer was left uncured prior to cementation.

A dual-cure resin cement (Duo-Link; Bisco, Schaumburg, IL, USA) was used to lute the inlays under firm pressure until fully seated. Excess cement was removed with a probe and microbrush. The restoration margins were coated with glycerin gel and light-cured for 40 seconds from four directions, after which all margins were polished. All bonding and restorative steps were performed by a single trained operator.

### Thermomechanical aging and FS test

**Fig. 3:**
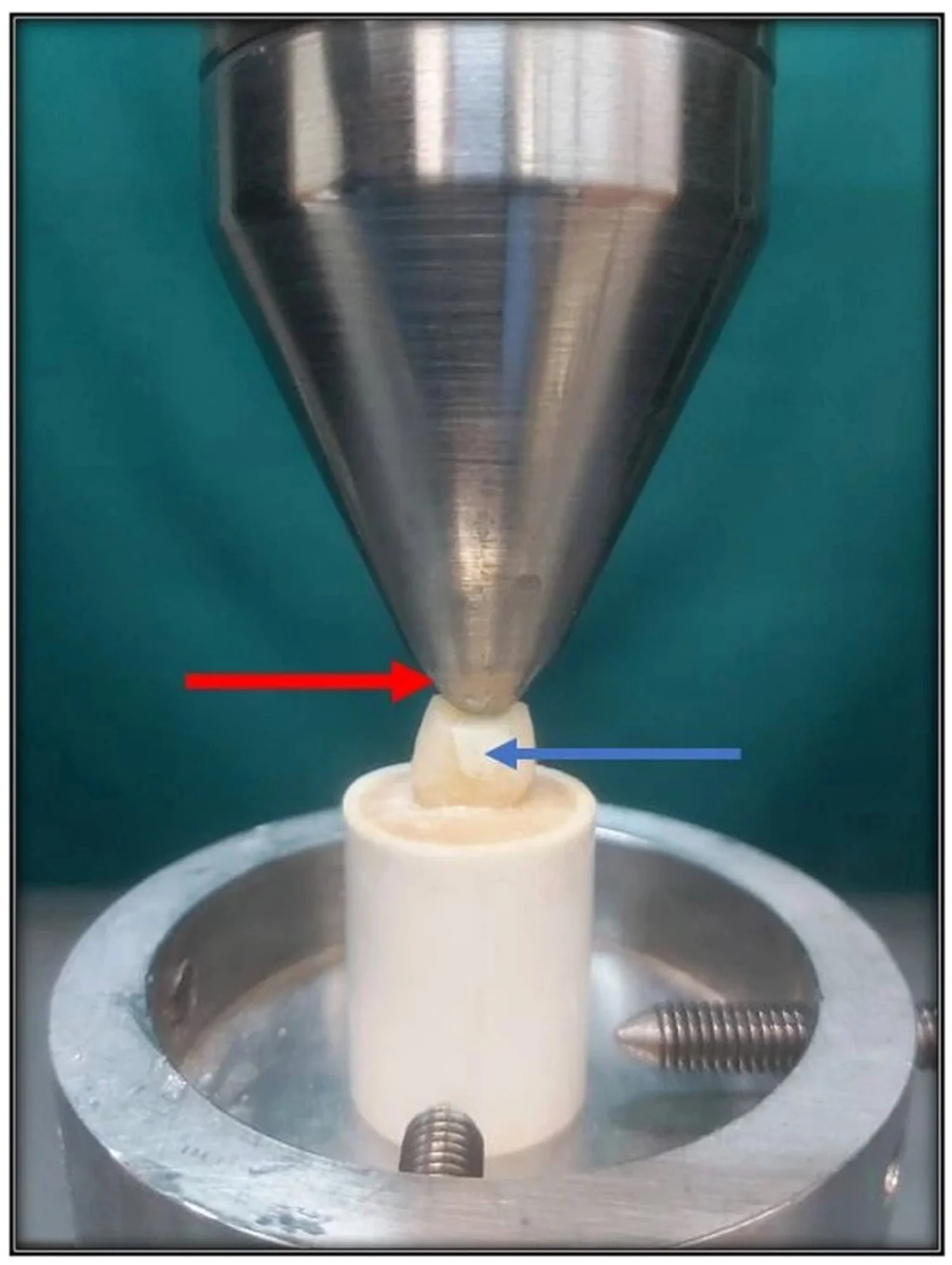
Mechanical testing assembly for fracture resistance. The restored specimen is shown mounted in the universal testing machine for compressive strength evaluation. The red arrow indicates rounded stainless-steel loading tip positioned against the occlusal surface of the restoration. The blue arrow highlights the ceramic inlay restoration in situ.

All cemented inlays were subjected to 100,000 loading cycles of 50 N at a frequency of 0.5 Hz in a mastication simulator (Chewing Simulator CS4; SD Mechatronic, Feldkirchen-Westerham, Germany) using a rounded stainless-steel antagonist contacting both cusp ridges in a water environment.^25^ Thermocycling was performed to simulate thermal stresses occurring in the oral environment and to provide a standardized aging protocol for in vitro evaluation of restorative materials. Although no universally accepted conversion exists between thermocycling cycles and clinical service time, previous studies have suggested that 5000-10000 cycles are commonly used to simulate clinical aging, depending on experimental conditions. Therefore, 8,000 thermocycles were used in the present study as an intermediate aging protocol within this commonly applied range.^11^^,^^26^ After aging, specimens were mounted in a universal testing machine (Instron Z020, Zwick Roell, Ulm, Germany) and subjected to axial compressive loading at a crosshead speed of 1 mm/min. The maximum load at failure was recorded in newtons (N) as the FS. Figure 3 illustrates a cemented inlay-restored specimen positioned in the universal testing machine for the fracture resistance test.

Fractured specimens were examined under a stereomicroscope at 40× magnification and categorized into two fracture types:

• Restorable: fractures confined above the CEJ
• Non-restorable: fractures extending below the CEJ into the root

**Fig. 4:**
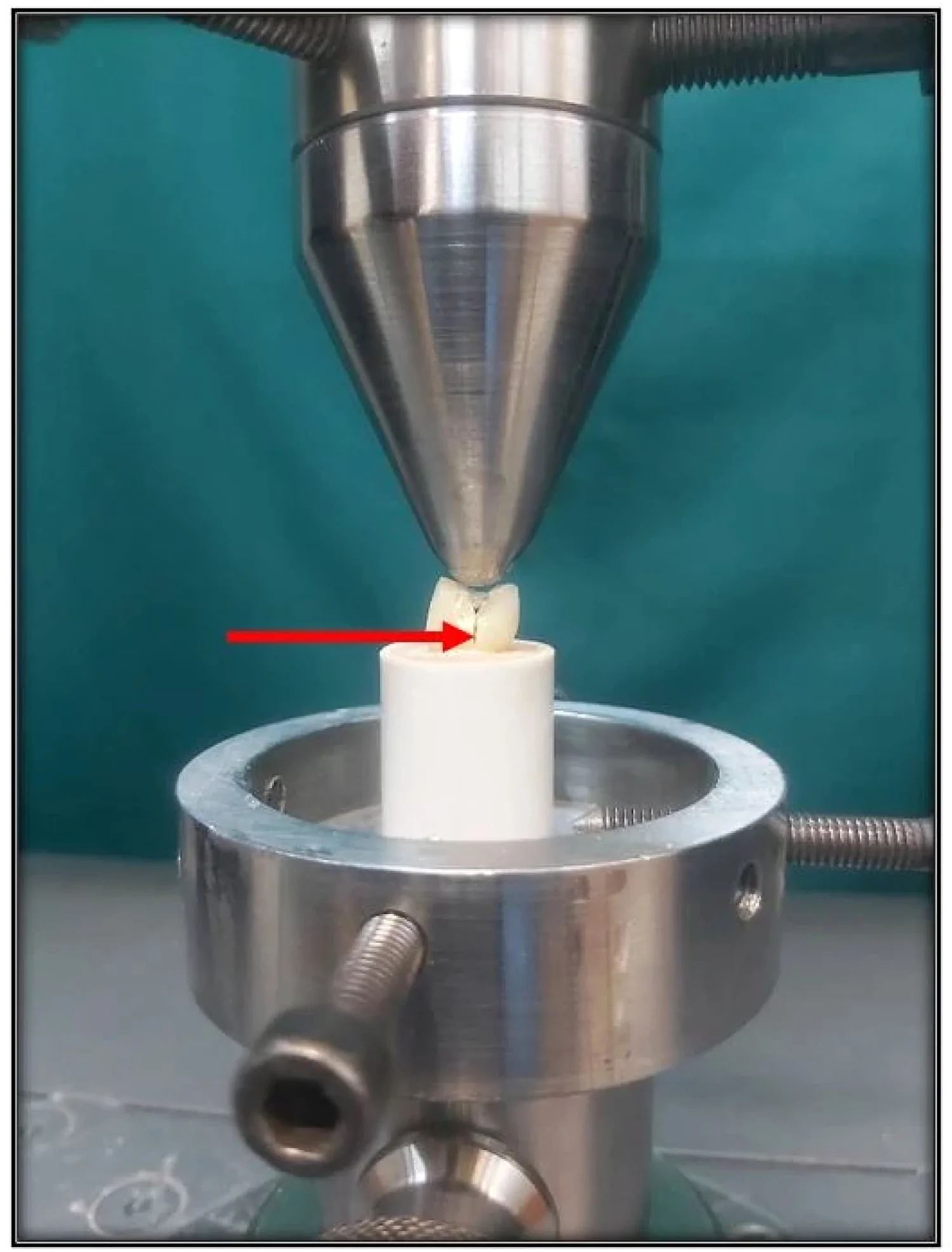
Failure mode visualization during fracture resistance testing. A representative image of a specimen at the point of catastrophic failure under compressive loading. The red arrow points to the propagation of a longitudinal fracture line through the tooth structure and the ceramic restoration. This pattern exemplifies a 'non-restorable' failure, extending beyond the cemento-enamel junction (CEJ). The stainless-steel loading tip is shown in contact with the occlusal surface at the moment of fracture.

Figure 4 demonstrates a tested specimen under the universal testing machine after experiencing a non-restorable fracture during the fracture resistance evaluation.

### Statistical analysis

Statistical analysis was performed using SPSS version 17 (SPSS Inc., Chicago, IL, USA). Normality of data distribution was assessed using the Kolmogorov–Smirnov test, and homogeneity of variances was evaluated using Levene’s test. A three-way analysis of variance (ANOVA) was used to examine the effects of diabetes status (diabetic vs non-diabetic), sealing technique (IDS vs DDS), and quercetin treatment (with vs without Qu), as well as their interaction effects, on fracture strength (FS). Effect sizes were reported as partial eta squared ($\eta_{p}^{2}$) to estimate the magnitude of the observed effects, and 95% confidence intervals were calculated for the group means. Following ANOVA, a limited number of predefined pairwise comparisons, specified a priori according to the study hypotheses and factorial design, were conducted using independent-samples t-tests. Because these comparisons were planned in advance and were not exploratory post hoc analyses, no formal adjustment for multiple testing (eg, Bonferroni or Holm–Bonferroni correction) was applied. The significance level was set at *P* < .05.

## Results

The mean FS values and standard deviations (SD), together with the 95% confidence intervals, are presented in Table 1. To complement the inferential findings, effect sizes were calculated and reported as partial eta squared (ηp²) for all main effects and interactions in the ANOVA model.

**Table 1:** Mean fracture strength (FS) values (mean ± standard deviation and 95% confidence interval) of ceramic inlay–restored premolars according to diabetic status, sealing technique, and quercetin treatment (n = 12 per group).

Tooth Type Technique/Treatment	Non-diabetic, mean ± SD (95% CI)	Diabetic, mean ± SD (95% CI)
DDS	724.83 ± 92.195 (665.9-783.8)	545.17 ± 71.747 (499.3-591.1)
DDS/Qu	756.33 ± 92.874 (696.9-815.8)	696 ± 91.042 (637.8-754.2)
IDS	960.33 ± 82.662 (907.4-1013.3)	712.58 ± 96.708 (650.7-774.5)
IDS/Qu	1048 ± 82.632 (995.1-1100.9)	891.58 ± 75.631 (843.2-940.0)

DDS, delayed dentin sealing; IDS, immediate dentin sealing; Qu, quercetin CI, confidence interval.

The three-way ANOVA showed significant main effects of diabetes status, sealing technique, and Qu treatment on FS (all *P* < .001) (Table 2), with large effect sizes for diabetes status (ηp² = 0.229) and sealing technique (ηp² = 0.338), and a moderate effect size for Qu treatment (ηp² = 0.168). Significant interactions were found between diabetes status and technique (F = 5.510, *P* = .021, ηp² = 0.061) and between diabetes status and Qu treatment (F = 8.912, *P* = .004, ηp² = 0.099). The interaction between technique and Qu treatment was not significant (F = 1.468, *P* = .229, ηp^2^ = 0.015), nor was the three-way interaction among all factors (F = 0.168, *P* = .683, ηp² = 0.002).

**Table 2:** Three-way ANOVA results showing the effects of diabetes status, dentin sealing technique, quercetin treatment, and their interactions on fracture strength.

Source	SS	df	Mean square	F	*P* value	Partial η²
DM	624037.500	1	624037.500	84.204	<.001*	0.229
Technique (DDS/IDS)	1190821.500	1	1190821.500	160.683	<.001*	0.338
Qu treatment	303525.042	1	303525.042	40.956	<.001*	0.168
DM × Technique	40837.500	1	40837.500	5.510	.021*	0.061
DM × Qu treatment	66045.042	1	66045.042	8.912	.004*	0.099
Technique × Qu treatment	10880.042	1	10880.042	1.468	.229	0.015
DM× Technique × Qu treatment	1247.042	1	1247.042	0.168	.683	0.002
Error	652166.167	88	7410.979	-	-	-
Total	63100568.00	96		-	-	-

ANOVA, analysis of variance; DM, diabetes mellitus; DDS, delayed dentin sealing; IDS, immediate dentin sealing; Qu, quercetin; SS, sum of squares; df, degrees of freedom; F, F statistic; η², eta squared.

⁎ Statistically significant at *P* < .05.

To further examine the predefined comparisons of interest, independent t-tests were performed within the specified subgroups. In the DDS, IDS, and IDS/Qu groups, diabetic teeth demonstrated significantly lower FS values than non-diabetic teeth (all *P* < .001). In contrast, no significant difference was observed between diabetic and non-diabetic teeth in the DDS/Qu group (*P* = .122).

Because the technique × Qu interaction was not significant, the planned pairwise comparisons indicated that IDS produced significantly higher FS values than DDS in both diabetic and non-diabetic teeth (both *P* < .001). Qu treatment significantly increased FS in diabetic teeth (*P* < .001), whereas it had no significant effect in non-diabetic teeth (*P* = .202) (Table 3).

**Table 3:** Subgroup comparison of fracture strength values (mean ± SD) according to dentin sealing technique and quercetin treatment in diabetic and non-diabetic premolars (n = 24 per subgroup).

Variable		Non-diabetic (mean ± SD)	Diabetic (mean ± SD)
Technique	**DDS**	740.58 ± 91.901	620.58 ± 111.180
	**IDS**	1004.58 ± 92.610	802.08 ± 124.768
	***P* value**	<.001*	<.001*
Treatment	**No treatment**	824.58 ± 147.640	628.88 ± 119.359
	**Qu treatment**	902.58 ± 172.365	793.77 ± 129.147
	***P* value**	.202	<.001*

DDS, delayed dentin sealing; IDS, immediate dentin sealing; Qu, quercetin.

⁎ *P* values < .05 are considered significant.

The distribution of fracture patterns across all experimental groups is summarized in Table 4, indicating the frequency of restorable fractures (above the CEJ) and non-restorable fractures (extending below the CEJ). The distribution of restorable and non-restorable fractures among the experimental groups was analyzed using Fisher’s exact test, which showed no statistically significant difference (p=0.112).

**Table 4:** Fracture mode of restored premolars (n = 12 per group).

Tooth Type Technique/Treatment	Non-diabetic (Restorable / Non-restorable)	Diabetic (Restorable / Non-restorable)
DDS	3/9	5/7
DDS/Qu	5/7	4/8
IDS	8/4	8/4
IDS/Qu	8/4	9/3

DDS, delayed dentin sealing; IDS, immediate dentin sealing; Qu, quercetin.

Restorable: fracture above the CEJ; Non-restorable: fracture extending below the CEJ.

## Discussions

DM is a chronic metabolic disorder, and current estimates indicate that approximately one in every eleven adults worldwide lives with this condition.^27^ Its prevalence continues to rise, with projections suggesting that more than 700 million individuals will be affected by 2040. Among the two major forms of the disease, type 2 DM accounts for roughly 81% to 92% of all cases.^28^

Ensuring the long-term effectiveness of restorative procedures in diabetic patients is therefore of particular clinical relevance. Recent investigations have examined the influence of DM on the bonding performance of adhesive systems to enamel and dentin.^14^^,^^29^^,^^30^ These studies consistently reported a negative short-term impact of diabetes on adhesive bond strength.^14^^,^^29^^,^^30^ Notably, many of these in vitro experiments did not include thermocycling and dynamic loadings, which are factors known to significantly affect the durability of resin–dentin bonds. In contrast, the FS test used in the present study more closely reflects clinical conditions, incorporating variables such as the configuration factor (C-factor) associated with inlay preparations, cavity compliance, and cyclic masticatory loading. Previous biomechanical investigations have also demonstrated that restorative materials can significantly influence stress redistribution within compromised tooth structures and improve fracture resistance by restoring near-physiological stress patterns.^31^ Chewing stress, in particular, has been linked to accelerated collagen degradation.^32^

Regarding clinical feasibility, the combination of IDS and Qu application adds additional steps to the restorative workflow, potentially increasing chairside time and technique sensitivity. IDS may improve the integrity of the resin–dentin interface and contribute to better stress distribution within the restored tooth structure. This increased complexity may be associated with improved adhesive performance, as reflected by the higher fracture strength after thermomechanical aging; however, direct evaluation of hybrid-layer longevity was not performed in this study. Qu acts as a potent cross-linker and MMP inhibitor; by stabilizing the collagen matrix and preventing enzymatic degradation, it addresses one of the primary causes of bond failure over time. In a clinical setting, the brief application of a Qu-rich solution during the IDS phase can be seamlessly integrated into the bonding protocol, offering a proactive approach to enhancing the durability of indirect restorations in high-risk groups, such as diabetic patients, where dentin quality may be compromised. Similarly, recent studies have highlighted the growing interest in bioactive materials aimed at preserving and regenerating dental tissues.^33^ For example, Naga et al. (2025) reported that an alginate hydrogel loaded with boron-doped mesoporous bioactive glass nanoparticles promoted dentin bridge formation and maintained pulp vitality in a canine model, further supporting the potential of biologically driven strategies to improve dental tissue outcomes.^33^

Previous work has also shown that maintaining a bonded enamel margin helps safeguard the underlying resin–dentin interface from degradation.^34^ Consequently, the concurrent stability of enamel and dentin bonds during thermomechanical aging may explain the fracture behavior observed in both diabetic and non-diabetic inlay-restored teeth.

The findings of this study demonstrated that conventional inlay cementation after a two-week temporization period (DDS) resulted in significantly lower FS in diabetic teeth compared with non-diabetic ones following thermomechanical aging. The ANOVA results further indicated that sealing technique had the largest effect on FS, followed by diabetes status, while Qu treatment also exerted a meaningful influence. In contrast, the observed interaction effects were smaller in magnitude, suggesting that the primary factors contributed more substantially to FS than their combined effects. The 95% confidence intervals further supported the consistency of these findings, particularly the superior performance of IDS and the reduced FS observed in diabetic specimens. These findings are consistent with previous research reporting compromised bonding performance of etch-and-rinse (E&R) adhesives to dentin, and even to both dentin and enamel, in diabetic conditions, with the effect being more pronounced in type 1 DM.^14^^,^^29^^,^^30^ Moreover, type 2 DM has been shown to exert a greater detrimental influence on enamel bonding than on dentin.^30^ Such concurrent reductions in enamel and dentin bonding efficacy may partially explain the decreased FS observed in the diabetic DDS group.

One contributing factor is the upregulated activity of MMPs (MMP-2 and MMP-9) associated with hyperglycemia and oxidative stress in diabetic patients.^17^ These enzymes promote collagen fibril degradation, undermining the long-term durability of the adhesive interface.^29^ This issue is especially pronounced with E&R adhesives, where incompletely infiltrated collagen within the demineralized dentin remains vulnerable. Additionally, the chewing-like stresses applied in the present study might further intensify collagen breakdown, amplify interfacial deterioration and diminish the reinforcing effect expected from adhesive luting in the diabetic DDS group.

Interestingly, applying Qu to the etched dentin during final cementation raised the fracture resistance of diabetic teeth to a level comparable with non-diabetic specimens. This improvement is likely related to the well-documented anti-MMP effects of Qu which can help limit collagen degradation within the hybrid layer.^19^^,^^20^ Moreover, Qu’s collagen-cross-linking capability may enhance the mechanical strength of the demineralized dentin substrate, contributing to greater interfacial stability.

Although Qu treatment improved the performance of diabetic teeth, it did not produce any measurable change in fracture resistance in the non-diabetic group, thereby partially supporting the null hypothesis. This discrepancy may reflect fundamental differences in the nanostructural and biomechanical properties of diabetic versus healthy dentin.^16^ Diabetes has been shown to interfere with normal calcification processes, leading to reduced mineral deposition. Peritubular dentin appears particularly affected by this altered mineralization, resulting in diminished peritubular dentin formation along with increased dentinal tubule diameter and higher tubular density.^13^^,^^14^^,^^16^ These changes are likely more pronounced in the deeper dentin involved in an inlay preparation.

Hyperglycemia-induced glycation also affects dentinal collagen, making it stiffer and more brittle.^14^ These alterations, together with reductions in trace elements such as strontium, may adversely influence dentin microhardness.^16^ When combined with cyclic loading, such changes may increase the susceptibility of dentin to crack formation or fracture.^16^ In addition, the structurally compromised nature of diabetic dentin, characterized by increased dentinal tubule density, may further contribute to the reduced fracture resistance observed in diabetic teeth.^13^ A similar explanation has been proposed for the lower fracture resistance of diabetic roots and their greater susceptibility to vertical fracture under occlusal forces.^35^ The possible similarity between the effects of DM on dental tissues and bone has also been reported.^14^^,^^35^ Diabetes has been shown to influence bone formation, metabolism, and fracture susceptibility.^36^ Placing restorations on a weakened dentin substrate has been associated with premature degradation and possible displacement of the restoration.^30^ In this context, Qu may play a beneficial role. It has been reported to improve the elastic modulus of dentin and facilitate the formation of a stable hybrid layer between tooth structure and adhesive materials.^37^ Furthermore, Qu has a relatively low molecular weight and exhibits both hydrophobic and hydrophilic characteristics due to its planar aromatic nucleus and polar hydroxyl groups, respectively.^19^^,^^20^ These properties may enhance the penetration of ABU adhesive resin into dentinal tubules that are wider, more numerous, and contain higher water content. Collagen cross-linking agents have also been shown to influence the water dynamics of dentin by resisting collagenase activity and reducing the formation of water channels within the collagen matrix.^21^^,^^37^ As a result, the overall water content of dentin may decrease, which could facilitate improved resin infiltration and polymerization.^38^ Collectively, these mechanisms may explain the positive effect of Qu pretreatment on the fracture resistance of diabetic teeth.

Some authors have reported no improvement in the bonding stability of caries-affected dentin, possibly because temperature fluctuations and pH reduction can alter its molecular structure.^39^ In the present study, the non-diabetic teeth likewise did not show any benefit from Qu application. These inconsistent findings may be explained by differences in dentin substrates, variations in experimental protocols, and the specific adhesive systems used across studies.

The other cementation approach examined in this investigation was IDS. In both diabetic and non-diabetic groups, IDS increased the fracture resistance of inlay-restored teeth, which led to partially rejecting the null hypothesis. Consistent with these findings, several previous studies have reported that IDS can enhance the fracture resistance of ceramic inlay restorations.^10^^,^^11^ The same positive effect was observed in diabetic teeth in the current study. This improvement may be attributed to the higher adhesive strength achieved through IDS, which provides better support for brittle ceramic restorations. Increased bond strength and reduced long-term degradation following IDS have been documented in earlier research.^7^^,^^8^^,^^40^^,^^41^ Nonetheless, some authors have questioned the long-term advantages of IDS, reporting limited improvements in bond strength and marginal adaptation after aging.^42^^,^^43^

Considering the structural challenges associated with bonding to diabetic dentin, the immediate establishment of the adhesive interface on freshly prepared diabetic dentin appears to be advantageous for improving fracture resistance.

In the four IDS groups of the present study, a recently described modification of the IDS protocol, referred to as reinforced IDS, was applied. This approach involves placing an additional layer of flowable resin over simplified adhesive systems. The flowable coating reinforces and protects the hybridized dentin layer and helps prevent re-exposure of dentinal tubules during the removal of temporary cements.^24^ This resilient intermediate layer may also act as a stress-absorbing component, reducing stresses generated by polymerization shrinkage of the resin cement in inlay cavities with a relatively high C-factor.^44^ Such an effect may partly explain the higher fracture resistance observed in all IDS groups compared with the DDS groups in the present study. This mechanism may also account for the greater proportion of restorable fractures observed in the IDS groups in both diabetic and non-diabetic specimens, although the differences in fracture mode distribution among the groups were not statistically significant. In addition, the fracture-mode analysis was performed across eight experimental groups with a limited number of specimens per group, which may have reduced the statistical power to detect differences in the distribution of restorable and non-restorable fractures. Future studies with larger sample sizes may benefit from pooled or stratified analyses based on clinically relevant factors, such as sealing technique and quercetin treatment.

In this study, ABU was applied using the E&R mode. However, some clinicians prefer to avoid acid etching of deep dentin and instead use the self-etch approach because it is simpler and faster to apply, provides greater resiliency, and may reduce postoperative sensitivity.^7^ Therefore, further investigations are required to evaluate the performance of the IDS technique when ABU is used in the self-etch mode.

In the intraoral environment, reduced salivary flow, diminished buffering capacity, lower pH, and increased plaque accumulation associated with DM can lead to higher enzymatic activity compared with non-diabetic individuals. Diabetic patients, particularly those with type 2 DM, also tend to harbor slightly higher levels of oral bacteria.^45^ As a result, the adhesive interface becomes more vulnerable to degradation and, over time, more prone to recurrent caries. This situation underscores the need for incorporating antibacterial and anti-MMP agents, such as Qu and other polyphenols, at the adhesive interface to enhance the long-term stability of adhesive restorations. The strong inhibitory effect of Qu against S. mutans and its ability to limit biofilm formation have been previously demonstrated.^21^ Nevertheless, it should be noted that this in vitro study did not fully replicate in vivo conditions, including the presence of vital pulp.

In addition to statistical significance, the clinical relevance of the observed FS values should be considered. Reported maximum occlusal forces in premolar regions have been estimated to range approximately between 350 and 450 N in healthy individuals, depending on parafunctional activity and measurement conditions.^46^ In the present study, mean FS values of all experimental groups exceeded this range, suggesting that all restorative approaches provided resistance beyond typical functional occlusal loads. Notably, IDS and Qu-treated groups demonstrated significantly higher FS values compared with their counterparts, which may indicate an increased safety margin under functional loading conditions. However, it should be emphasized that in vitro fracture testing does not fully replicate the complexity of intraoral forces, fatigue loading, or long-term degradation processes, and therefore clinical extrapolation should be made with caution.

The results of this study should be interpreted with caution, as the experimental design represents an in vitro model using extracted teeth, simulated periodontal ligament, and limited aging conditions. Important factors present in the oral environment, including pulpal pressure, oral biofilm, and long-term functional loading, were not reproduced and may influence the clinical performance of restorative procedures. Furthermore, although the subgroup comparisons were predefined according to the study hypotheses and factorial design, no formal correction for multiple testing was applied. Consequently, a potential increase in the risk of Type I error should be considered when interpreting the results of these comparisons.

Another limitation relates to the verification of diabetic status. The selection of teeth from diabetic patients was based on self-reported medical history and verbal confirmation of a diagnosis of diabetes mellitus for at least five years. Objective biochemical verification, such as HbA1c or fasting blood glucose values, was not available because many participants did not undergo regular six-month evaluations and lacked consistent laboratory documentation. Since the duration and severity of hyperglycemia may influence systemic complications and potentially affect the mechanical properties of the dentin–adhesive interface, the inability to assess glycemic control should be considered when interpreting the findings. Therefore, the results should not be generalized to all diabetic patients without considering variations in glycemic status.

In addition, the sample size calculation was originally based on a previously published two-group comparison rather than on the three-factor ANOVA design used in the present study. Although the significant two-way interactions and their corresponding effect sizes suggest that the study was adequately powered to detect moderate interaction effects, the post hoc analysis indicated limited statistical power for detecting the three-way interaction among diabetes status, sealing technique, and Qu treatment. Therefore, the absence of a significant three-way interaction should be interpreted with caution.

Furthermore, although the diabetic and non-diabetic donors were selected from a comparable age range (35-55 years), age-related variations in dentin structure and mechanical properties may still have influenced the results and therefore represent a potential limitation of the study. Future long-term clinical trials incorporating various anti-MMP and antibacterial agents at the adhesive interface are recommended to better guide clinicians in selecting optimal restorative strategies for diabetic patients undergoing adhesive cementation procedures.

## Conclusion

Within the limitations of this in vitro study, the findings suggest that both the reinforced IDS technique and Qu application, individually and in combination on acid-etched dentin prior to the application of ABU, may improve the FS of inlay-restored premolars in diabetic specimens. In non-diabetic specimens, an increase in FS was observed primarily with the IDS technique. The highest FS values in both diabetic and non-diabetic groups were recorded when IDS was combined with Qu treatment. These findings should be interpreted with caution, as this in vitro design does not fully replicate clinical conditions. Although Qu’s reported anticaries and anti-MMP properties may suggest a potential benefit, long-term clinical studies are required to confirm its effect on restoration longevity.

## Author contributions

*Conceptualization*: All authors. *Data curation*: All authors. *Formal analysis*: Fereshteh Shafiei, Zahra Jowkar. *Funding acquisition*: Fereshteh Shafiei, Zahra Jowkar. *Investigation*: Fereshteh Shafiei, Zahra Jowkar. *Methodology*: All authors. *Project administration*: Fereshteh Shafiei, Zahra Jowkar. *Resources*: Fereshteh Shafiei, Zahra Jowkar. *Software*: Fereshteh Shafiei, Zahra Jowkar. *Supervision*: All authors. *Validation*: Fereshteh Shafiei, Zahra Jowkar. *Visualization*: Fereshteh Shafiei, Zahra Jowkar. *Roles/Writing - original draft*: Fereshteh Shafiei, Zahra Jowkar. *Writing - review & editing*: Fereshteh Shafiei, Zahra Jowkar. *Writing - review*: Mahshid Mohammadi-Bassir.

## Funding

This research (Number:34196) was financially supported by Shiraz University of Medical Sciences, Shiraz, Iran.

## Data availability

Data available on request from the author.

## Conflict of interest

None disclosed.
